# Laminar N-Doped Carbon Materials from a Biopolymer for Use as a Catalytic Support for Hydrodechlorination Catalysts

**DOI:** 10.3390/ma14113107

**Published:** 2021-06-05

**Authors:** Cristina Ruiz-Garcia, Miguel A. Gilarranz

**Affiliations:** 1 Chemical Engineering Department, Faculty of Sciences, Universidad Autónoma de Madrid (UAM), Cantoblanco, 28049 Madrid, Spain; cristina.ruiz@ciemat.es; 2 CIEMAT, Av. Complutense 40, 28040 Madrid, Spain

**Keywords:** N-doped carbon, salt templating, catalysis, palladium, hydrodechlorination

## Abstract

Nitrogen-doped porous carbons were prepared using a chitosan biopolymer as both a carbon and nitrogen precursor and metallic salts (CaCl_2_ and ZnCl_2_-KCl) as a templating agent with the aim of evaluating their performance as catalyst supports. Mixtures of chitosan and templating salts were prepared by simple grinding subjected to pyrolysis and finally washed with water to remove the salts. The resulting materials were characterized, showing that homogeneous nitrogen doping of carbon was achieved (7–9% wt.) thanks to the presence of a nitrogen species in the chitosan structure. A lamellar morphology was developed with carbon sheets randomly distributed and folded on themselves, creating slit-shaped pores. Substantial porosity was observed in both the micropore and mesopore range with a higher surface area and microporosity in the case of the materials prepared by ZnCl_2_-KCl templating and a larger size of mesopores in the case of ZnCl_2_. Catalysts with well-dispersed Pd nanoparticles (around 10 nm in diameter size) were synthesized using the chitosan-based carbons obtained both by salt templating and direct chitosan pyrolysis and tested in the aqueous phase hydrodechlorination of 4-chlorophenol. The fast and total removal of 4-chlorophenol was observed in the case of catalysts based on carbons obtained by templating with CaCl_2_ and ZnCl_2_-KCl in spite of the low metal content of the catalysts (0.25% Pd, wt.).

## 1. Introduction

**Figure 1:**
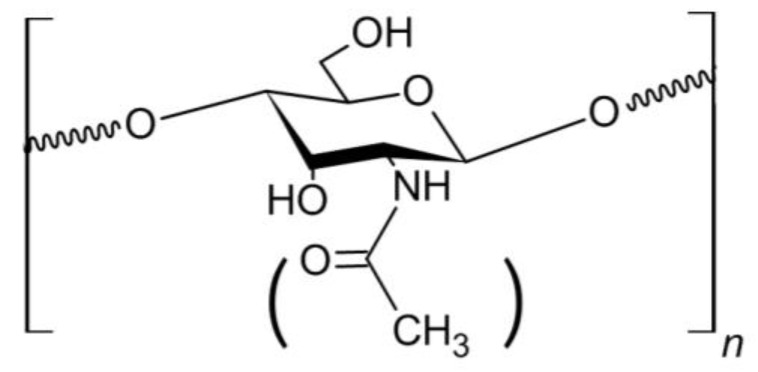
Chitosan structure.

Nitrogen-doped carbon materials have been extensively used in the last years in a large number of applications including catalysis, separation and adsorption, electrode materials in supercapacitors, batteries or sensing, among others [1]. The main properties that make these materials attractive are their thermal and chemical stability, which allow their use in a wide temperature range even in the presence of aggressive reactants. There is a wide variety of reported procedures to prepare N-doped carbon materials although most of them are based on two main approaches. The first of them is the modification of a starting carbon with doping agents where the control of the doping level is generally low and doping agents that are expensive and dangerous are usually involved [2] and the second is the direct pyrolysis of nitrogen-rich starting materials where the nitrogen precursor can be a component of the carbon precursor or an additive. Polymers (polyacrylonitrile, polyanilines, etc.) and organic and inorganic molecules (amino acids, ammonia, urea, etc.) have been extensively used for this purpose [3]. Lately, biopolymers such as chitosan have taken an important role because of the search for more sustainable processes [4]. Chitosan is derived from the partial deacetylation of chitin, one of the most abundant biopolymers, which shows repeated units of N-acetyl-D-glucosamine and D-glucosamine (Figure 1). The use of chitosan as a precursor of N-doped carbon has been reported for the preparation of different carbon-based materials such as graphene films, carbon dots [5], amorphous particles, spongy nanoflakes morphologies [6] or lamellar and porous structures [7]. The control of the ultimate structure depends on the pretreatment of the precursor mixtures, the procedure during the thermal treatment and the solvents and activating agents employed. An important aspect to consider in the preparation of carbon materials is the development of a well-defined porous structure, enabling materials to be exposed to a high surface area and thus improving the interface contact [6,8]. This feature, which favors a mass transfer through the carbon material, is highly demanded in a number of applications. In particular, it is key in the mechanisms involved in catalytic applications and more specifically in heterogeneous catalysis. In this sense, different procedures have been described to obtain porous carbons with a controlled structure including hard templating, which involves porous silicas and zeolites as templates [9]. This method involves the use of corrosive and hazardous solvents to remove the silica templates and generates waste streams that pose a risk, which is a serious drawback. Alternatives have been explored; hence, metallic salts [10,11,12], carbonates [13] and hydroxides [14] have been postulated as good candidates to develop a well-defined porosity in the carbon matrix, being easily removed with hot water or weakly acidic solutions. The synthesis process uses carbonates, hydroxides, nitrates and chlorates and proceeds through the partial oxidation of the starting carbon, producing CO that contributes to the activation [15]. In the case of metallic salts such as NaCl, CaCl_2_ or ZnCl_2_, the process has been referred to as the “salt templating” method [10,16,17]. The carbon precursor, the carbonizate and the salts are immiscible, which leads to porous carbons with a pore structure derived from the salt clusters and their percolation structures. The election of the salts determines the pore size of the final carbon; thus, the development of mesopores is characteristic of salt templating using CaCl_2_ [18] whereas ZnCl_2_ generates microporosity or mesoporosity depending on whether it is used alone or together with another salt [10]. The capacity of a few precursors such as chitosan to form chelates due to the strong coordination of nitrogen groups with metal ions has also been found to contribute to the activation [19]. Likewise, the procedure for mixing and crushing the precursors and salts has a role in the morphology and porous structure of carbon [7].

The role of carbon support N-doping in the performance of catalysts has been studied in previous works. A few studies on hydrodechlorination reported that N-doping of carbon supports leads to an improvement in the activity of the catalysts; however, the role of the nitrogen species is not fully understood. Basic sites in N-doped carbons have been found to provoke an increase in support polarity and carbon atoms adjacent to N have a higher capacity as electron donors [20,21]. In addition to this, a few of the N-functional groups created such as pyridinic and pyrrolic can lead to an increase in energy binding and the stability of active phase nanoparticles [22,23,24]. Likewise, an enhanced adsorption of chlorophenols on N-doped carbons has been observed, which can contribute to the reaction pathway [25].

Previous works have proved that the performance of an N-doped carbon catalyst in hydrodechlorination processes is highly dependent on support properties [25] such as N content and the porous surface. In this context, in the current study, N-doped carbons from chitosan were prepared using metallic salts as templates. The materials obtained were characterized and were evaluated as a catalyst support in the hydrodechlorination of 4-chlorophenol (4-CPh) in an aqueous phase as a model reaction. 

## 2. Materials and Methods

Solid mixtures of a low molecular weight chitosan (Sigma–Aldrich, Madrid, Spain) carbon precursor and metal salts were prepared by solid-state grinding in an agate mortar to achieve intimate mixing. The used salts were anhydrous CaCl_2_, KCl and ZnCl_2_ provided by Panreac (Madrid, Spain). Mass ratios for ZnCl_2_:KCl, chitosan:CaCl_2_ and chitosan:ZnCl_2_-KCl of 59:61, 1:1 and 1:3 were chosen based on a previous bibliography [18]. The ground chitosan:salt mixtures were pyrolyzed at 700 °C after fast heating under a nitrogen flow of 30 NmL·min^−1^. After 1 h of reaction time, the samples were cooled to room temperature under a nitrogen flow. Subsequently, the pyrolyzed samples were ground in a mortar, washed with water to eliminate the remaining salts and dried in an oven at 60 °C. The nitrogen-doped carbons obtained from the mixtures of chitosan and CaCl_2_ and ZnCl_2_:KCl were denoted as C/Ca and C/Zn-K, respectively. Carbons were obtained also by the direct pyrolysis of chitosan and denoted as C_blank_. These carbons were used as a support of Pd catalysts, which were prepared by incipient wetness impregnation in the case of C/Ca and C/Zn-K and by wetness impregnation in the case of C_blank_, yielding catalysts denoted as Pd-C/Ca, Pd-C/Zn-K and Pd-C_blank_, respectively. The impregnation of the carbon supports was carried out with a solution of palladium chloride in HCl of 0.1 M. The impregnated solids were dried at 65 °C overnight, calcined at 200 °C for 2 h and reduced at 100 °C in an H_2_ atmosphere, giving rise to catalysts with a nominal Pd load of 0.25% (wt). 

The textural properties of the carbons obtained were studied from the nitrogen adsorption-desorption isotherms at 77 K and the carbon dioxide adsorption isotherm at 273 K obtained in Tristar II 3020 equipment (Micromeritics, Norcross, GA, USA) after degassing at 130 °C for at least 4 h under a vacuum (~0.1 mbar). The surface area was calculated by the Brunauer–Emmet–Teller (BET) equation and the external area was calculated using the t-plot method. The micropore and mesopore volumes and pore size distributions were calculated by the t-plot and BJH methods, respectively, and also by NLDFT calculations. The morphology was studied by transmission electron microscopy (TEM) with a JEM 2100 microscope (JEOL, Tokyo, Japan). Chemical elemental analyses were performed in a LECO CHNS-932 apparatus (St Joseph, MI, USA). X-ray photoelectronic spectroscopy (XPS) experiments were recorded on a PHI VersaProbe II apparatus (Physical Electronics, Chanhassen, MN, USA) using monochromatic Al-Kα (1486.6 eV) radiation and a dual beam charge neutralizing system. The spectra of the materials were recorded using a 100 µm beam size operating at 25 W and 15 kV and using a pass energy of 29.35 eV. MultiPak 9.3 software (Multiscience GmbH, Hoya, Germany) was employed to calculate the semi-quantitative elemental surface analyses and to deconvolute the spectra of the C 1s, N 1s and O 1s regions. The processing of the collected spectra was performed referencing the energy binding to the C 1s peak located at 284.6 eV. The available palladium surface was characterized by CO chemisorption at room temperature (PulseChemiSorb 2705, Micromeritics, Norcross, GA, USA) to determine metal dispersion assuming a Pd−CO stoichiometry of 1 [26]. X-ray diffraction patterns were recorded between 3–60 (2θ) degrees in a diffractometer (Bruker D8 Discover, LYNXEYE detector, Bruker, Billerica, MA, USA) equipped with Cu Kα (0.15406 nm) radiation. The structural parameters were deduced by applying Scherrer’s equation *L* = (*K∙λ*/*β∙cosθ*) where L corresponds with the average height of the crystal stacking (H), K being a dimensionless factor with a value of 0.9 for a 002 reflection, λ is the wavelength of the X-rays, β is the width of the diffraction signal in radians and θ is the Bragg angle.

The 4-CPh hydrodechlorination experiments were carried out at 30 °C in a glass stirred batch reactor using 0.150 L of a 0.1 g L^−1^ 4-CPh aqueous solution, 2.4 mg_Pd_ L^−1^, 50 NmL min^−1^ H_2_ flow and constant stirring (800 rpm). Homogeneous samples of around 1.0 mL were collected at different reaction times (0, 5, 15, 30 min and 1, 2 and 4 h). The samples were filtered and analyzed by HPLC with a UV-Vis detector (Prostar, Varian Inc., Palo Alto, CA, USA) using a mixture of acetonitrile and water 1:1 as carrier phase and a C18 column, and by GC-FID (GC 3900 Varian Inc., Palo Alto, CA, USA) using a capillary column (CP-Wax 52 CB, Varian) and N_2_ as a carrier gas. 

A pseudo-first-order rate equation was used to fit the experimental data for a 4-CPh disappearance. To compare the catalysts tested, the catalytic activity (*a*) and turnover frequency (TOF) were calculated as *a* = *k* · *C*_0_/*w* and *TOF* = (*k* · *C*_0_ · *N_A_* · *S_M_*)/(*w* · *SSA_M_*), respectively, where *C*_0_ represents the starting molar concentration of 4-CPh, *w* is the metal active phase concentration expressed as g_Pd_·L^−1^, *S_M_* is the surface of a metal atom, *SSA_M_* is the surface of the metal and *k* is the pseudo-first-order rate constant obtained for the disappearance of 4-CPh.

## 3. Results and Discussion

### 3.1. Characterization Materials

The chemical composition of the carbon materials obtained is shown in Table 1. The starting polymer exhibited an important amount of oxygen and hydrogen; however, more than ca. 75% of these elements were eliminated during the reactions involved in pyrolysis. Enrichment in carbon was observed for all of the synthetized carbons with a few differences in the carbon and nitrogen content among the samples.

**Figure 2:**
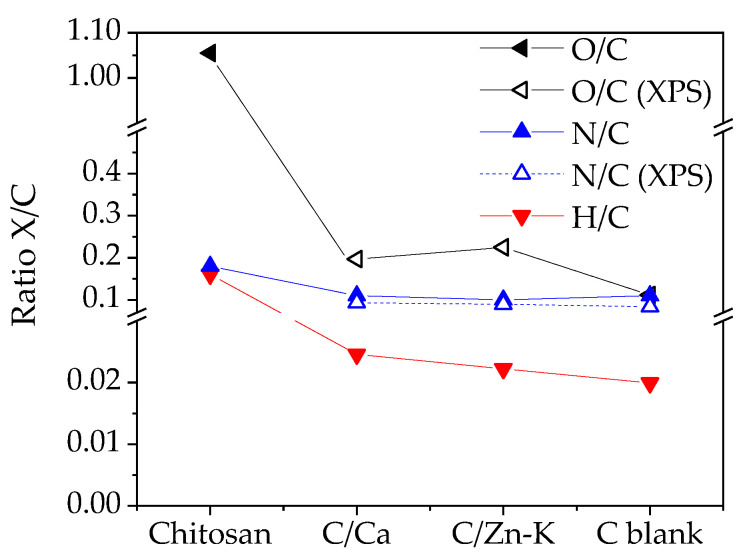
O/C, H/C and N/C ratios for the carbon materials prepared.

Figure 2 shows the H/C, O/C and N/C ratios and helps to understand the conversion of the starting chitosan through the thermal treatment under different conditions, i.e., the pyrolysis in the presence of different metallic salts. The decrease in the O/C ratio upon pyrolysis was relevant for all samples although it was slightly higher for C/Ca and C/Zn-K carbons than for the C_blank_ sample, showing that metallic salts not only acted as an activating agent but also favored the generation of oxygenated functional groups. It has to be noted that the O/C ratios calculated for C_blank_ from the chemical and XPS analysis were similar, indicating a homogeneous distribution of oxygen groups in the material. However, there was uncertainty on whether the higher O/C ratio obtained by XPS for C/Ca and C/Zn-K was partly due to the enrichment of the surface oxygen groups at the most superficial layers of the material. The higher generation of the functional groups for these samples could be related also with the higher H/C ratios observed for them although the high decrease for all samples in relation to starting chitosan showed a high extent of dehydrogenation reactions [27] and consequently an increase in aromaticity [28]. As in the case of oxygen, the close N/C ratio values obtained by the chemical and XPS analysis might indicate a homogeneous distribution of N in the carbon materials. 

The carbon, oxygen and nitrogen species in the carbon materials were identified by the deconvolution of the corresponding XPS spectra (Figures S1–S3), as shown in Table 2. The species identified from the C 1s spectra show equivalent percentages for all carbons although a higher contribution of C sp3 in C_blank_ should be noted. Regarding the groups deduced from the O 1s peak deconvolution, i.e., quinones, carbonyl (C=O), ethers/hydroxyl (C-O) and carboxyl/esters, they showed similar magnitude for all samples. The higher atomic proportions for carbons C/Ca and C/Zn-K, except in the case of the O-C=O group, could be attributed to the effect of the metallic salts. Regarding the nitrogen groups, i.e., pyridinic, pyrrolic, quaternary and pyridinic oxide and N oxides, no relevant differences among them could be found in the set of the carbons studied, with similar pyridinic/quaternary ratios also. The similarity in the functional groups could be attributed to the use of the same carbon source, being only modified slightly due to the presence of the metallic salts.

**Figure 3:**
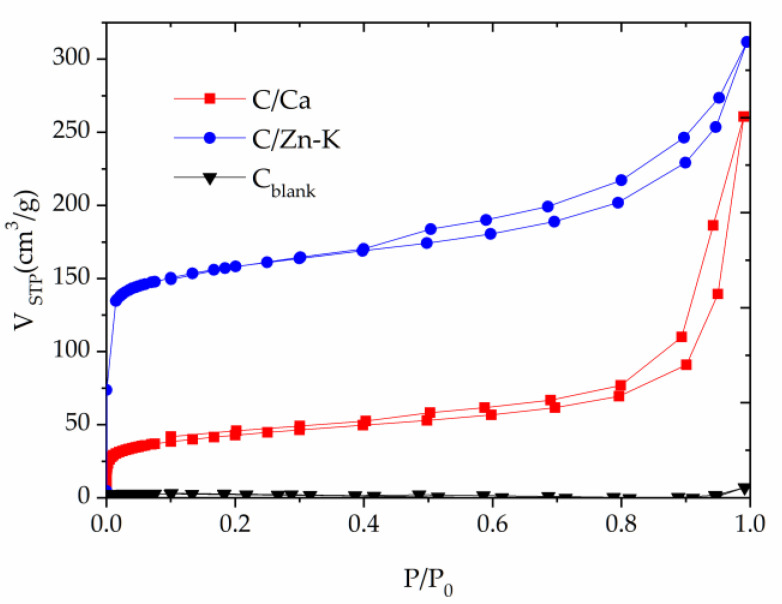
Nitrogen adsorption-desorption isotherms at 77 K for the carbon materials prepared.

The nitrogen adsorption-desorption isotherms at 77 K shown in Figure 3 revealed important differences among the carbon materials. The uptake in the case of C_blank_ was very low; however, the uptake for the materials obtained when metallic salts were used indicated a relevant development of porosity. C/Ca and C/Zn-K exhibited type I/II isotherms at low and high relative pressures, respectively. A relevant uptake and sharp knee at a low relative pressure evidenced the development of microporosity. The uptake was higher for C/Zn-K, which also showed a wide hysteresis loop for P/P_0_ above 0.4 that could be considered as H3 and was usually related to capillary condensation in slit- and wedged-shaped porosity mesopores resulting from particle aggregates. The presence of Zn salts during pyrolysis has been found to catalyze the depolymerization and decomposition of carbohydrates leading to an increase in porosity [29]. The broader hysteresis loop in the case of C/Ca also indicated a wider range of mesopore size. The textural properties calculated from the adsorption-desorption isotherms are shown in Table 3. The values of BET and an external surface area of 605 and 197 m^2^/g were obtained for sample C/Zn-K, being 153 and 102 m^2^/g for C/Ca. The main difference in the pore volume could be attributed to more developed microporosity in the case of C/Zn-K. As expected from the isotherm, C_blank_ showed a very low BET and external surface area, characteristic of a virtually non-porous material. However, CO_2_ isotherms obtained at 273 K evidenced the presence of ultramicropores. The CO_2_ isotherms showed minor differences among the carbon materials prepared regarding the ultramicropore volume. This characteristic of the pore texture could be associated with the carbonizate formed directly from chitosan and not related to the presence of metal salts during pyrolysis. 

**Figure 4:**
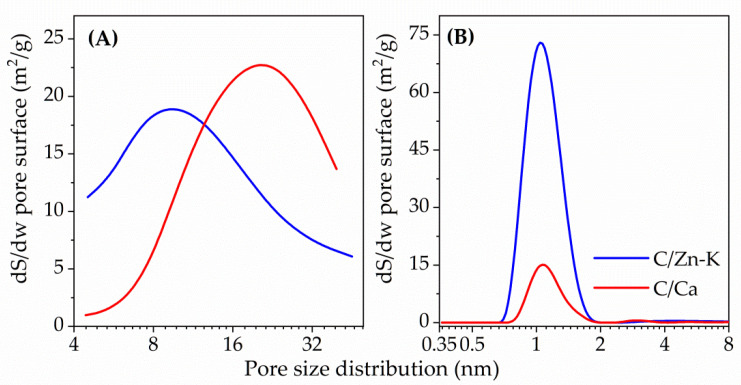
Pore size distribution from N_2_ adsorption-desorption isotherms for: (**A**) mesopore range as calculated by BJH and (**B**) micropore range as calculated by NLDFT.

The pore size distribution obtained by NLDFT from the N_2_ adsorption-desorption isotherm centered around 1 nm for both C/Ca and C/Zn-K (Figure 4). The mesopore size distribution calculated by BJH theory showed a broad range for both samples and also significant differences, being centered around 10 nm for C/Zn-K and 21 nm for C/Ca. Therefore, the carbon materials obtained presented a similar chemical composition but differed in porous texture. 

**Figure 5:**
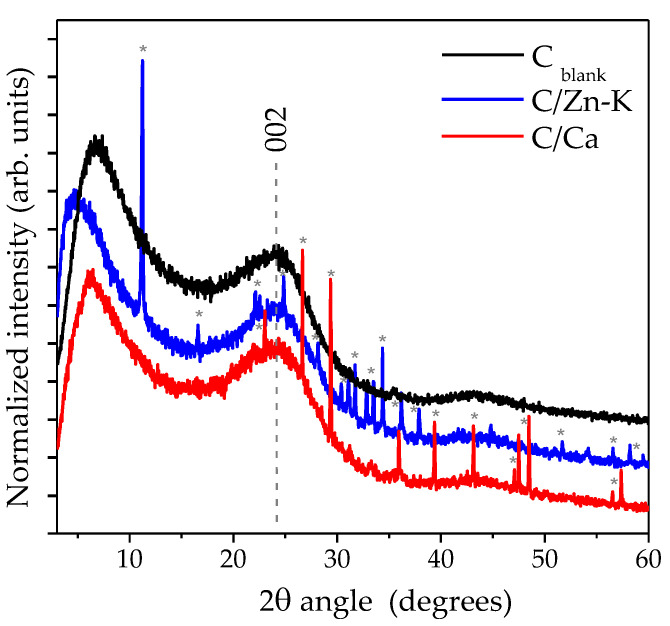
XRD patterns of carbon materials prepared normalized to a 002 reflection. Reflections from the remaining metallic salts are noted as (*).

The X-ray diffractogram patterns (Figure 5) exhibited two intense but broad reflections, which could be related to an absence of structural order, probably owing to the distortions induced by the fast pyrolysis treatment of the chitosan and also to the presence of the N heteroatoms in the carbon lattice. There was a reflection at ca. 0.39 nm distance (23° of 2θ angle) attributed to the graphitic-like phase formed after the thermal treatment, ascribed to the (002) plane. The other relevant reflection was centered at lower angles, between 5 and 7°, corresponding with 1.77 and 1.26 nm. The origin and the meaning of this reflection was unclear; however, several studies in the literature have shown that a few carbon materials as zeolite-templated carbons or graphenic layers folded on themselves can exhibit reflections at low angles of 2θ. Zeolite-templated carbons display an XRD pattern at low angles comparable with the materials under study, which were attributed to the structural pore ordering transferred from the starting zeolite [30]. Likewise, other authors described in a graphenic layered material a reflection at low angles of 2θ (11.8°) that was related to the distance of the channel formed at the folding axis in the graphene layers [31,32]. This reflection in the diffractogram of C_blank_ suggested that short range domains with a higher structural ordering may result from the carbonization of chitosan upon fast pyrolysis. The size of the crystal domains calculated from the (002) reflection is shown in Table 4, revealing similar values for all of the samples; around 0.7–0.9 nm for the average height (H). The XRD patterns of C/Ca and C/Zn-K exhibited several strong and sharp reflections with a high crystallinity owing to the salt residues in the material. The XRD results could be interpreted as being indicative of low structural ordering of the carbons obtained.

**Figure 6:**
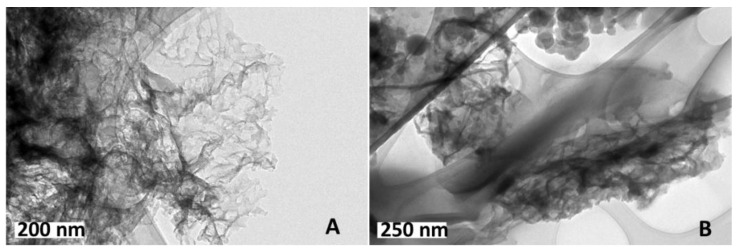
Representative micrographs obtained by TEM of (**A**) C/Ca and (**B**) C/Zn-K.

The low structural order evidenced by the XRD patterns was coherent with the micrographs obtained by TEM (Figure 6), where the carbon samples C/Ca and C/Zn-K could be observed to have a corrugated shape of a thin lamella folded on themselves in agreement also with the slit and wedge shape of the pores inferred from the N_2_ adsorption-desorption isotherms. In the case of C/Zn-K, a smoother surface and the occurrence of aggregates could also be observed. Similar structures were described by Gao et al. [33], who prepared carbons with different morphologies from chitin (an acetylated form of chitosan) using different heating rates. High heating rates resulted in greater shrinkage and crimped carbon materials as a consequence of fast dehydration and pyrolysis reactions leading to C-C or C-O bond cleavages [34]. Primo et al. [35] used chitosan as a starting material to obtain N-doped graphene and observed good quality layers when low heating rates were used during pyrolysis. 

CO chemisorption was employed to calculate the Pd dispersion and nanoparticle mean size for the catalysts synthesized using the carbons prepared from chitosan (Table 5). Both Pd-C/Zn-K and Pd-C/Ca showed dispersions around 11% and a diameter size of 9–10 nm; however, in the case of the Pd-C_blank_ a lower dispersion was obtained with a Pd nanoparticle size larger than 100 nm. The low specific surface area of C_blank_ might be related to the growth of such large Pd particles. The metal nanoparticle size for Pd-C/Zn-K was only slightly smaller than for Pd-C/Ca. It has to be noted that the metal load was very low (0.25 % wt.) and that both carbon supports exhibited a wide availability of O and N surface groups that could serve as nucleation and anchoring sites. Therefore, the surface available seemed to be enough to achieve a good dispersion. The difference in S_BET_ between C/Zn-K and C/Ca corresponded essentially with microporosity as could be seen from the N_2_ and CO_2_ adsorption isotherms. Previous works in the literature have shown that microporosity, particularly narrow microporosity, may be hindered for carbons rich in surface groups thus diminishing the liquid phase metal adsorption [36]. Accordingly, it has been observed that the formation of nanoparticles within micropores is low in the case of carbons with a high surface group content [37].

### 3.2. Catalytic Activity Results

**Figure 7:**
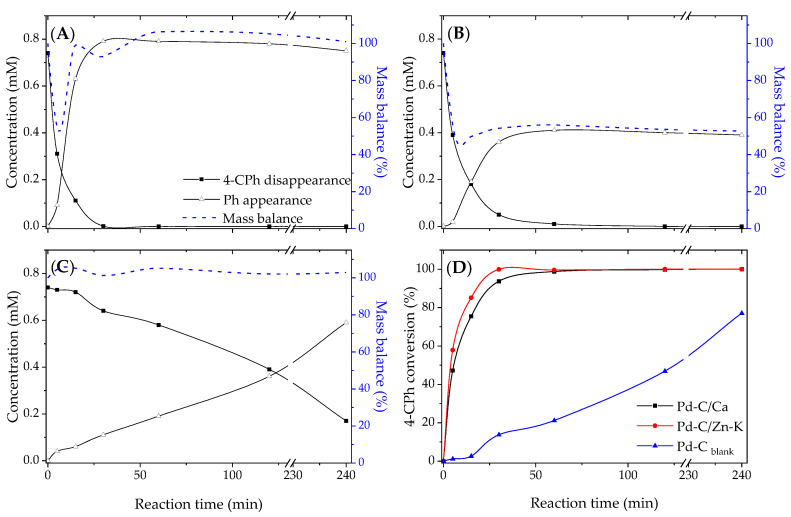
Evolution of the hydrodechlorination reaction and mass balance closure using (**A**) Pd-C/Ca, (**B**) Pd-C/Zn-K, (**C**) Pd-C_blank_ catalysts and (**D**) a 4-CPh conversion in the catalytic tests.

The catalysts synthesized using the carbon materials prepared from chitosan were tested in 4-CPh hydrodechlorination as proof of the concept of the features that the biomass starting material could confer to catalysts. The reaction was carried out in a batch aqueous phase under a hydrogen flow as indicated in the Materials and Methods Section. Figure 7 shows the species concentration and mass balance for the initial (0–120 min) and long (230–240 min) reaction times. Although all of the catalysts were active, they yielded different 4-CPh disappearance rates and final conversions, as can be observed in Figure 7. In the case of catalysts Pd-C/Ca and Pd-C/Zn-K, the fast disappearance of 4-chlorophenol was observed, reaching a total 4-CPh conversion after 30 and 60 min reaction times, respectively. A substantially slower disappearance was observed for Pd-C_blank_, which only achieved ca. 75% conversion after 240 min. These important differences in activity could be ascribed primarily to the size of Pd nanoparticles in the catalyst. In the case of catalysts Pd-C/Ca and Pd-C/Zn-K, the nanoparticle size was similar; therefore, the wider mesoporosity of the support could contribute to the higher overall rate. The disappearance of 4-chlorophenol could be considered as relevant taking into account the very low metal content (0.25 % wt.) and the Pd nanoparticle size of the catalysts. An additional improvement in activity could be achieved in the tuning dispersion by a Pd precursor selection [38].

**Figure 8:**
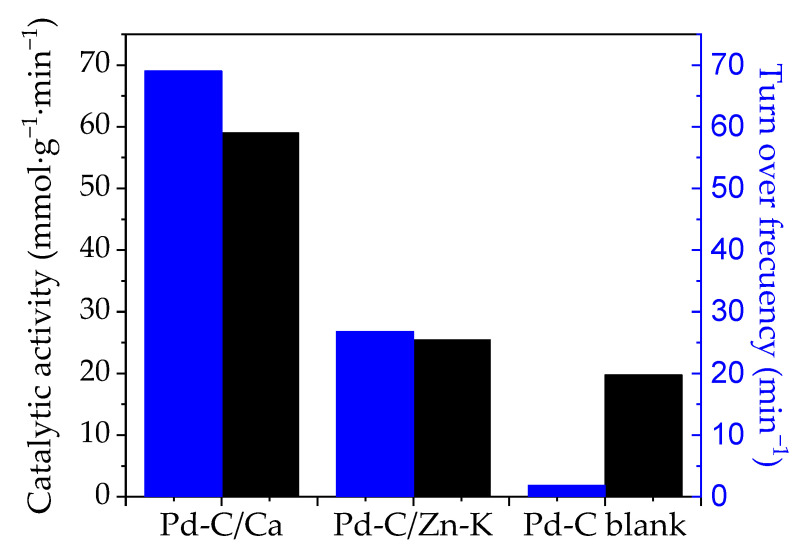
Comparison of the catalytic activity and the TOF values for the catalysts studied.

Interestingly, Pd-C/Ca showed both a faster disappearance of 4-CPh and a very high mass balance closure throughout the reaction test (Figure 8). However, the mass balance for the reaction test catalyzed by Pd-C/Zn-K only closed around 50%, indicating an important adsorption of the species involved in the reaction. In addition to phenol, hydrogenation products such as cyclohexanone and cyclohexanol were detected, although at trace levels, in agreement with the reaction mechanism reported elsewhere [39]. Regarding the presence of the nitrogen groups on the catalyst support, an amount of influence in the catalytic activity could be expected. However, relevant differences in the type of nitrogen groups and their abundance were not observed between C/Ca and C/Zn-K, as discussed above. In a previous work [40], the catalytic activity in a hydrodechlorination reaction reached values below 5 mmolg^−1^_Pd_ min^−1^ using a reduced graphene oxide doped with nitrogen as a Pd support with a comparable chemical composition but with a lower surface area than the C/Ca and C/Zn-K, implying the differences in the activity capacity of the catalyst observed could be ascribed to the porous texture.

The catalytic activity was evaluated in terms of the TOF in order to take into account not only the kinetic constant for the disappearance but also the Pd nanoparticle size, thus showing catalytic cycles taking place at the available Pd per unit time (Table 6). Differences in the TOF values for Pd-C_blank_ and Pd-C/Zn-K were relatively small, 20 and 25 min^−1^, respectively, whereas the TOF was as high as 59 min^−1^ for Pd-C/Ca. The specific catalytic activity, i.e., per mass of metal, showed very low values for Pd/C_blank_ (2 mmol·g^−1^_Pd_·L^−1^) because of a low dispersion. The highest specific activity was obtained for the Pd/C-Ca catalyst, showing a value of 69 mmol·g^−1^_Pd_·L^−1^.

## 4. Conclusions

The synthesis of chitosan-based nitrogen-doped carbons was achieved successfully by templating with CaCl_2_ and ZnCl_2_-KCl. A chemical elemental analysis and XPS evidenced a homogeneous distribution of the nitrogen species in the bulk carbon structure with C/N ratios ca. 0.1, which could be ascribed to the well-dispersed nitrogen groups present in the chitosan structure. The predominant nitrogen functions in the carbon were pyridinic and pyrrolic although a significant contribution of quaternary nitrogen was also observed. Carbons were obtained as lamella randomly arrayed and folded on themselves, creating an open structure of great interest for catalysis in the liquid phase. Different topologies were achieved depending on the templating salt employed. A substantial contribution of mesoporosity was observed with slit-shaped slit- and wedged-shaped mesopores as inferred from the nitrogen adsorption-desorption isotherms but the surface area and microporosity were higher in the CaCl_2_-templated carbon whereas a larger size of mesopores was obtained in the case of ZnCl_2_-KCl. The Pd catalyst prepared from chitosan-based carbons exhibited a good dispersion with a nanoparticle mean size around 10 nm. A very high activity was observed in all cases, even in the case of the catalyst supported on non-templated carbon with a low surface area (TOF 20 min^−1^), thus showing the role of nitrogen doping in activity promotion. Optimum results were obtained for catalysts supported on salt-templated carbons, exhibiting TOFs of 59 and 25 min^−1^ in the case of CaCl_2_ and ZnCl_2_-KCl, respectively.

## Figures and Tables

**Table 1:** Chemical and XPS elemental analysis of the carbon materials prepared (% wt.).

	Chemical Analysis							XPS Analysis				
Sample	C	H	N	S	O est. *	N/C	O est./C	C	N	O	N/C	O/C
C/Ca	72.5	1.8	8.1	0.02	-	0.11	-	76	8	16	0.1	0.2
C/Zn-K	70.6	1.6	7.4	0.05	-	0.10	-	75	6	18	0.1	0.2
C_blank_	82.8	1.6	9.4	0.03	6.1	0.11	0.1	84	7	9	0.1	0.1
Chitosan	41.7	6.7	7.5	0.05	44.0	0.18	1.0	-	-	-	-	-

* Calculated for chitosan and C_blank_ by difference and neglecting ash content.

**Table 2:** Relative abundance (%) of the different peaks assigned from the deconvolution of the C 1s, O 1s and N 1s core energy levels of the carbon materials prepared.

**C 1s (at %); Binding Energy (eV)**						
	C-C sp^2^	C-C sp^3^	C-O/C-N	C=O	O-C=O	π-π *
Sample	284.60	285.50 ± 0.1	286.25 ± 0.05	287.4 ± 0.1	288.80	290.65 ± 0.05
C/Ca	52.3	3.8	0.8	0.9	0.2	0.2
C/Zn-K	52.9	1.8	0.9	0.8	0.3	0.2
C_blank_	51.2	9.0	0.4	0.7	0.2	0.2
**O 1s (at %); Binding Energy (eV)**						
	Quinones	C=O	C-O	O-C=O	H_2_O	$\frac{\sum C=O}{\sum C-O}$
Sample	530.4 ± 0.1	531.6 ± 0.1	532.5 ± 0.1	533.6 ± 0.1	535	
C/Ca	2.1	3.8	4.2	1.9	0.4	1.0
C/Zn-K	2.9	5.1	4.9	1.7	0.0	1.2
C_blank_	1.2	1.7	2.3	1.5	0.5	0.8
**N 1s (at %); Binding Energy (eV)**						
	Pyridinic-N	Pyrrolic-N	Quaternary-N	Pyridinic-N ox.	N-oxides	Pyridinic/ Quaternary
Sample	398.3 ± 0.1	400.3 ± 0.1	401.2 ± 0.1	402.8	405	
C/Ca	2.9	2.8	1.0	0.4	0.6	0.5
C/Zn-K	1.8	2.4	0.7	0.3	0.5	0.6
C_blank_	2.3	2.1	0.9	0.3	0.5	0.5

* Ratio $\frac{\sum C=O}{\sum C-O}$ calculated from the summation of C=O (quinones and C=O) and C-O (C-O and O-C=O); pyridinic/quaternary ratio calculated from the pyridinic-N and quaternary-N.

**Table 3:** Pore texture of the materials obtained from nitrogen adsorption-desorption isotherms at 77 K.

	$S_{BET}$	$S_{Ext}$	$S_{eq_{CO_{2}}}$	$V_{{µ}_{CO_{2}}}$	$V_{{µ}_{t-plot}}$	$V_{meso_{BJH}}$	$V_{{µ}_{NLDFT}}$	$V_{meso_{NLDFT}}$	$V_{total_{NLDFT}}$
Sample	(m^2^/g)			(cm^3^/g)					
C/Ca	153	102	595	0.239	0.0213	0.377	0.0412	0.356	0.397
C/Zn-K	605	197	542	0.217	0.160	0.333	0.212	0.241	0.453
C_blank_	6	0	515	0.206	-	-	-	-	-

**Table 4:** Structural parameters obtained from the XRD patterns for the carbon materials prepared.

	2θ Angle (°)	FWHM (°)	D (nm)	H (nm)	Layer Stacking
C/Ca	22.9	8.9	0.39	0.9	2–3
C/Zn-K	23.6	12.3	0.38	0.7	1–2
C _blank_	22.8	8.7	0.39	0.9	2–3

**Table 5:** Pd dispersion of catalysts as calculated from CO chemisorption.

Sample	Dispersion (%)	Diameter Size (nm)
Pd-C/Ca	10.8	10
Pd-C/Zn-K	12.5	9
Pd-C_blank_	1.0	120

**Table 6:** Pseudo-first-order rate constant for the 4-CPh disappearance, the TOF and the specific catalytic activity.

Sample	k (min^−1^)	r^2^	TOF (min^−1^)	a (mmol g^−1^_Pd_ min^−1^)
Pd-C/Ca	0.2295	0.94	59	69
Pd-C/Zn-K	0.0895	0.995	25	27
Pd-C _blank_	0.0062	0.991	20	2

## Data Availability

Not applicable.

## Supplementary Materials

The following are available online at https://www.mdpi.com/1996-1944/14/11/3107/s1, Figure S1: Deconvolution of XPS of C 1s spectra; Figure S2: Deconvolution of XPS of N 1s spectra; Figure S3: Deconvolution of XPS of O 1s spectra.
