# Supplementary material for: Laminar N-Doped Carbon Materials from a Biopolymer for Use as a Catalytic Support for Hydrodechlorination Catalysts
Source: Materials (Basel). 2021 Jun 5;14(11):3107. doi: 10.3390/ma14113107 (PMC8215644; doi:10.3390/ma14113107)
Supplement: Supplementary file 1 [file materials-14-03107-s001.zip › materials-1240742-supplementary.pdf]

materials

MDPI

Supplementary Materials

Article

Laminar N-Doped Carbon Materials from a Biopolymer for Use as a Catalytic Support for Hydrodechlorination Catalysts

Cristina Ruiz-García [1,2] and Miguel A. Gilarranz [1,*]

[1] Chemical Engineering Department, Faculty of Sciences, Universidad Autónoma de Madrid (UAM), Cantoblanco, 28049 Madrid, Spain
[2] CIEMAT, Av. Complutense 40, 28040 Madrid, Spain; cristina.ruiz@ciemat.es
* Correspondence: miguel.gilarranz@uam.es

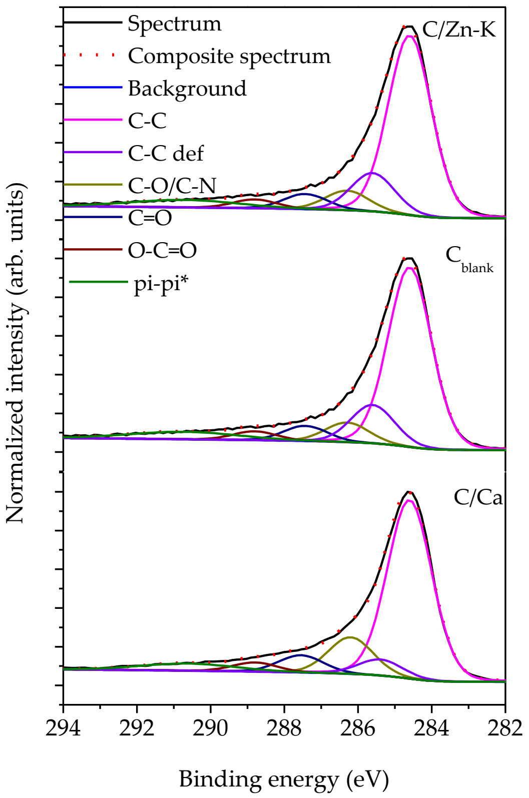

Figure S1. Deconvolution of XPS of C 1s spectra.

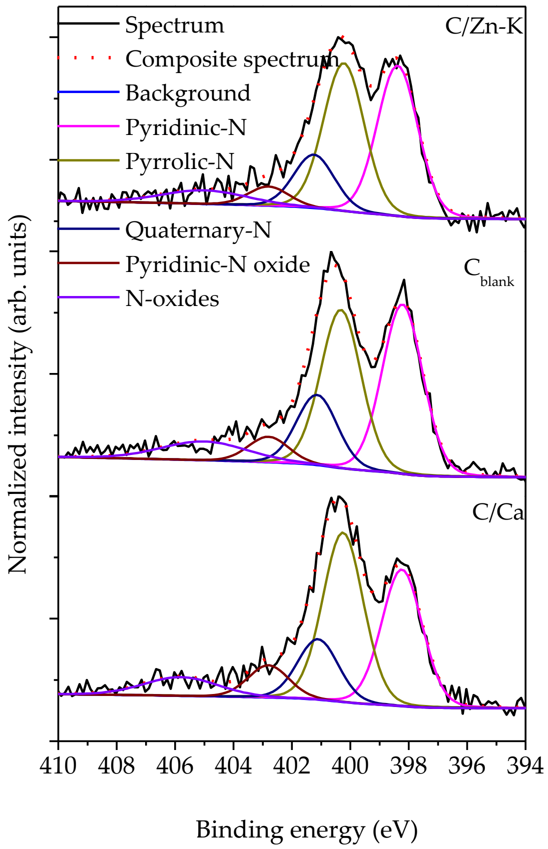

Figure S2. Deconvolution of XPS of N 1s spectra.

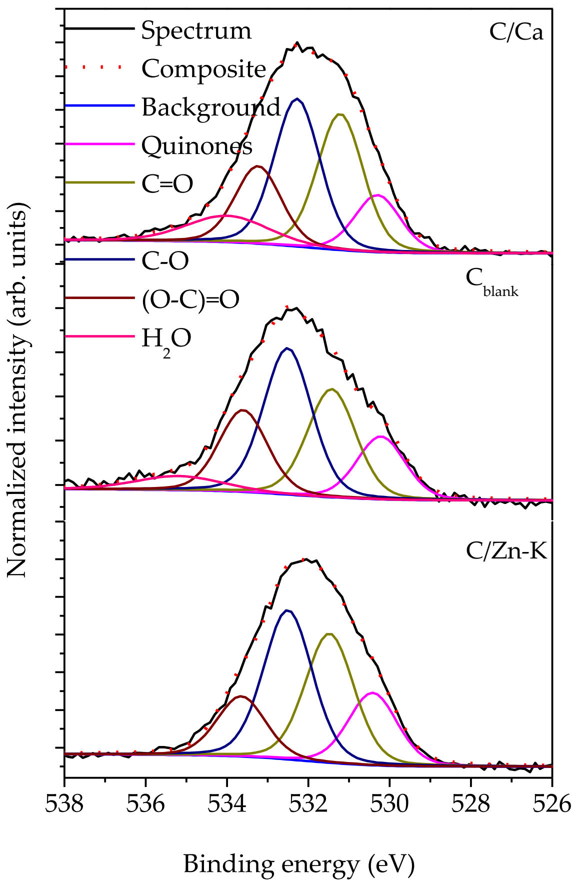

Figure S3. Deconvolution of XPS of O 1s spectra.
